# Hypoxia Reduces Arylsulfatase B Activity and Silencing Arylsulfatase B Replicates and Mediates the Effects of Hypoxia

**DOI:** 10.1371/journal.pone.0033250

**Published:** 2012-03-13

**Authors:** Sumit Bhattacharyya, Joanne K. Tobacman

**Affiliations:** 1 Department of Medicine, University of Illinois at Chicago, Chicago, Illinois, United States of America; 2 Department of Medicine, Jesse Brown VA Medical Center, Chicago, Illinois, United States of America; Fred Hutchinson Cancer Research Center, United States of America

## Abstract

This report presents evidence of 1) a role for arylsulfatase B (ARSB; N-acetylgalactosamine-4-sulfatase) in mediating intracellular oxygen signaling; 2) replication between the effects of ARSB silencing and hypoxia on sulfated glycosaminoglycan content, cellular redox status, and expression of hypoxia-associated genes; and 3) a mechanism whereby changes in chondroitin-4-sulfation that follow either hypoxia or ARSB silencing can induce transcriptional changes through galectin-3. ARSB removes 4-sulfate groups from the non-reducing end of chondroitin-4-sulfate and dermatan sulfate and is required for their degradation. For activity, ARSB requires modification of a critical cysteine residue by the formylglycine generating enzyme and by molecular oxygen. When primary human bronchial and human colonic epithelial cells were exposed to 10% O_2_×1 h, ARSB activity declined by ∼41% and ∼30% from baseline, as nuclear hypoxia inducible factor (HIF)-1α increased by ∼53% and ∼37%. When ARSB was silenced, nuclear HIF-1α increased by ∼81% and ∼61% from baseline, and mRNA expression increased to 3.73 (±0.34) times baseline. Inversely, ARSB overexpression reduced nuclear HIF-1α by ∼37% and ∼54% from baseline in the epithelial cells. Hypoxia, like ARSB silencing, significantly increased the total cellular sulfated glycosaminoglycans and chondroitin-4-sulfate (C4S) content. Both hypoxia and ARSB silencing had similar effects on the cellular redox status and on mRNA expression of hypoxia-associated genes. Transcriptional effects of both ARSB silencing and hypoxia may be mediated by reduction in galectin-3 binding to more highly sulfated C4S, since the galectin-3 that co-immunoprecipitated with C4S declined and the nuclear galectin-3 increased following ARSB knockdown and hypoxia.

## Introduction

Deficiency of the enzyme arylsulfatase B (ARSB; N-acetylgalactosamine-4-sulfatase) leads to the lysosomal storage disease mucopolysaccharidosis (MPS) VI (Maroteaux-Lamy-Syndrome), which is associated with accumulation of the sulfated glycosoaminoglycans chondroitin-4-sulfate (C4S) and dermatan sulfate (DS). In addition to lysosomal localization, ARSB is also present in the cell membrane of epithelial and endothelial cells [1]–[6]. The sulfatase enzymes are a family of enzymes that each have highly specified chemical function, and ARSB removes the 4-sulfate group from N-acetylgalactosamine-4-sulfate at the non-reducing end of C4S and DS, and thereby can regulate the degradation of these sulfated glycosaminoglycans (GAGs) [7]–[9]. The catalytic activity of ARSB requires post-transformational modification of a critical cysteine residue at position 91 by formylglycine modification, involving the formylglycine-generating enzyme (FGE) and molecular oxygen [10]–[14]. Other work has characterized the FGE as sulfatase modifying factor (SUMF)1 and addressed its migration from the endoplasmic reticulum, its secretion, and return to the endoplasmic reticulum [15]–[17]. Crystallization of ARSB demonstrated that the sterically favorable conformation of ARSB involves participation of a calcium ion to which the sulfate group from the C4 position of the N-acetylgalactosamine residue is bound [18], [19]. The critical cysteine residue of ARSB is restored following conversion by the FGE and molecular oxygen to a formylglycine (oxo-alanine) intermediate [10]–[14]. Specific correction of ARSB deficiency in MPS VI has been achieved by direct intravenous administration of recombinant human ARSB produced in CHO cells in which the post-translational formylglycine modification occurs [20], [21].

Our recent reports have indicated that ARSB activity is reduced in malignant colonic and mammary epithelial cells [1], [3], [22], [23] and in uncorrected cystic fibrosis bronchial epithelial cells [24]. Decline in ARSB activity has lead to reduced IL-8 secretion and increased IL-8 sequestration by bronchial epithelial cells [6] and increased cell-based kininogen and reduced bradykinin secretion by normal rat kidney epithelial cells [4]. Work of other investigators has shown the importance of chondroitin-4-sulfation in relation to attachment of malarial parasites to placental and vascular cells [25], [26], and the impact of chondroitin-4-sulfation on glial scar formation following spinal cord injury [27]. These studies demonstrate that ARSB, due to its essential role in the degradation of chondroitin-4-sulfate, has profound effects on a wide range of pathophysiological processes in multiple cell types and models.

The studies in this report were undertaken to better understand how ARSB activity and chondroitin-4-sulfation could influence such diverse and significant biological processes. In this report, the similar effects of hypoxia and of ARSB silencing in human bronchial and colonic epithelial cells in culture are presented for the first time. Since molecular oxygen is required for the post-translational modification of ARSB to its active form, we hypothesized that ARSB activity might be reduced in a hypoxic environment and that intracellular oxygen signaling might be mediated by ARSB. We considered if ARSB silencing could replicate the effects of oxygen on HIF-1α. Determinations of nuclear HIF-1α levels and mRNA expression were performed and supported the emerging concept that ARSB activation might serve as a cellular rheostat to regulate molecular oxygen signaling. To obtain additional evidence about the similarity of the effects of hypoxia and ARSB, the impact of hypoxia on the expression of 84 genes in a hypoxia signaling PCR array and on the levels of sulfated glycosaminoglycan (GAG) and chondroitin-4-sulfate (C4S) levels were determined. Recognizing that a mechanism by which the impact of ARSB effects on N-acetylgalactosamine-4-sulfation could be translated into transcriptional events was required, we proceeded to investigate the potential role of galectin-3 as an intermediary between changes in chondroitin sulfation and gene expression, based on the reported decline in galectin binding to more highly sulfated chondroitin sulfate [28]. Study findings that follow support galectin-mediation of signaling from environmental oxygen, through ARSB activation and chondroitin-4-sulfation, to nuclear AP-1-activation and enhanced HIF-1α transcription.

Also of interest was the consideration of how ARSB silencing and hypoxia might affect the impact of sulfate groups on cellular redox status, since sulfate assimilation is a well-recognized metabolic process in protists, plants, and yeast [29]–[31]. The sulfate assimilation pathway comprises a series of redox reactions by which sulfates are progressively reduced to sulfhydryls, including reduced glutathione and cysteine. In addition, sulfate groups participate in the sulfotransferase reactions that involve the 3′-phosphoadenosine 5′-phosphosulfate (PAPS), and thereby recycle into sulfated glycosaminoglycan synthetic processes and into sulfated storage substrates [32]–[36]. Measurements of total cellular sulfhydryl content and of the ratio of reduced glutathione to gluthatione disulfide were undertaken to assess if ARSB silencing and hypoxia both produced similar changes in the overall cellular redox status, consistent with inhibition of sulfate assimilation and reduced sulfhydryl production.

The experiments that follow in this report consider how ARSB mediates oxygen signaling; how changes in chondroitin-4-sulfation lead to transcriptional effects through changes in galectin-3 binding and galectin-3 nuclear translocation; and how the redox effects of oxygen and ARSB may result from effects on sulfate assimilation in human epithelial cells. We anticipate that further consideration of the role of sulfate in cellular metabolism will lead to improved understandings of the mechanisms that determine cell fate.

## Results

### Hypoxia reduces ARSB activity in human epithelial cells

**Figure 1:**
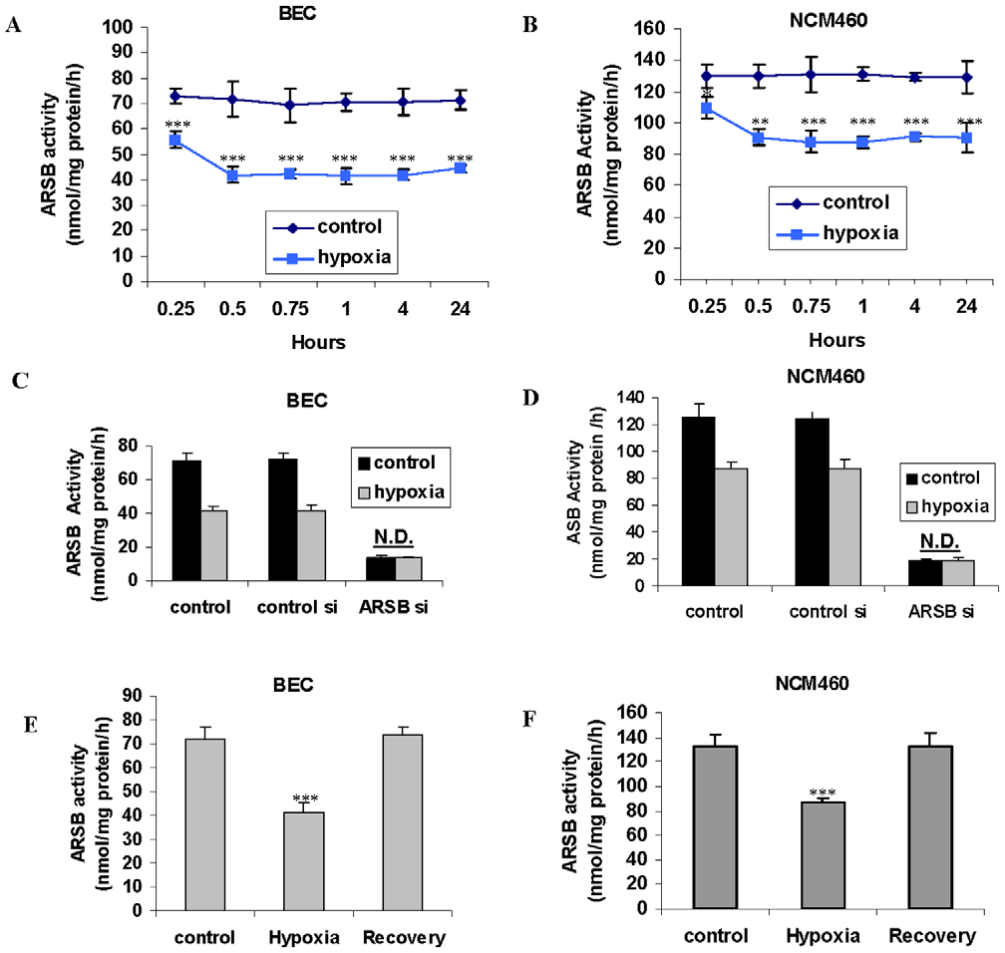
ARSB activity reduced by hypoxia in BEC and NCM460 cells. **A.** ARSB activity in the bronchial epithelial cells (BEC) was measured following exposure to 10% oxygen environment for 0.25, 0.5, 0.75, 1, 4, and 24 hours. ARSB declined significantly in activity by 0.25 hours (p<0.001), and remained at this level for 24 hours, in contrast to control cells under normoxic conditions. **B.** Similarly, following exposure to 10% O_2_, ARSB activity declined in the NCM460 cells and was significantly reduced at 0.25 h (p<0.05). ARSB declined further by 30 minutes (p<0.01), reaching maximum reduction by 0.75 h (p<0.001), and remained at this level for 24 h, in contrast to control cells. **C.** The combination of hypoxia (10% O_2_×4 h) and ARSB silencing in the BEC produced no further decline than that achieved by ARSB silencing alone. **D.** Similarly, in the NCM460 cells, the combination of ARSB silencing and hypoxia (10% O_2_×4 h) did not lead to further decline in the ARSB activity than that produced by ARSB siRNA alone. **E.** In the BEC, return to normoxia for 4 h after hypoxia (10% O_2_×4 h) restored the baseline ARSB activity. **F.** In the NCM460 cells, return to normoxia for 4 h after 4 h of 10% O_2_ restored the baseline ARSB activity. [ARSB = arylsulfatase B; BEC = bronchial epithelial cell; N.D. = no difference].

Since molecular oxygen is required for activation of ARSB, the effect of a reduced oxygen environment on ARSB activity was measured. Normal human bronchial epithelial cells (BEC) and human colonic epithelial cells (NCM460 cells) were exposed to a reduced oxygen environment (10%) for 15 minutes to 24 hours, and ARSB activity was determined using the exogenous substrate 4-methylumbilliferyl sulfate. PO_2_ declined 40–45% in the hypoxic NCM460 cells compared to the normoxic control cells at 1 h and 4 h. Maximum lowering of ARSB activity occurred by 1 h, declining to 57% (from 72.7±2.9 to 41.5±1.6 nmol/mg protein/h) and to 67% (from 130.0±7.8 to 87.8±3.7 nmol/mg protein/h) of the baseline activity, in the BEC (**Fig. 1A**) and NCM460 cells (**Fig. 1B**), respectively (p<0.001; 1-way ANOVA with Tukey-Kramer post-test). In contrast, activity of galactose-6-sulfatase (GALNS), steroid sulfatase (STS), or arylsulfatase A (ARSA) did not decline in the BEC or NCM460 cells following exposure to the reduced oxygen environment.

ARSB silencing by siRNA produced greater declines in ARSB activity than the 10% oxygen environment. ARSB declined 81% (from 70.7±6.7 to 13.5±1.0 nmol/mg protein/h) in the BEC and 85% (from 129.2±6.1 to 19.8±2.7 nmol/mg protein/h) in the NCM460 cells. The combination of hypoxia and ARSB silencing by siRNA did not produce any further decline in the ARSB activity than ARSB silencing alone in the BEC (**Fig. 1C**) or the NCM460 cells (**Fig. 1D**). Return to normoxia following exposure to hypoxia restored ARSB activity to baseline in the BEC (**Fig. 1E**) and NCM460 cells (**Fig. 1F**). Overexpression of ARSB increased activity to 175.6±9.4 nmol/mg protein/h in the BEC and to 230.4±10.7 nmol/mg protein/h in the BEC.

**Figure 2:**
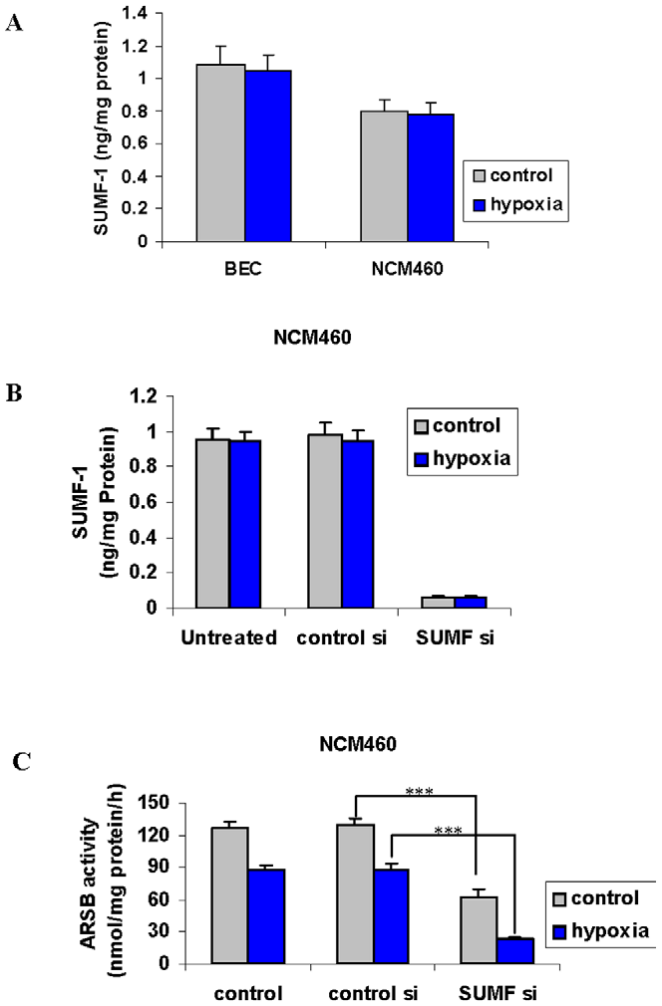
SUMF1 not modified by hypoxia in BEC or NCM460 cells. **A.** Protein expression of the sulfatase modifying factor (SUMF)-1 was not reduced by hypoxia (10% O_2_×4 h) in the BEC or the NCM460 cells. **B.** Knockdown by siRNA for SUMF-1 effectively reduced the protein expression measured by ELISA in the NCM460 cells. **C.** ARSB activity was significantly reduced by siRNA for SUMF-1 (p<0.001), and additional reduction occurred in association with hypoxia (10% O_2_×4 h) (p<0.001) in the NCM460 cells, consistent with a requirement for both SUMF-1 and molecular oxygen for maximum ARSB activity. [ARSB = arylsulfatase B; BEC = bronchial epithelial cell].

To rule out effects of hypoxia on the sulfatase modifying factor (SUMF)-1 as the explanation for reduced ARSB activity, SUMF-1 was measured in the BEC and NCM460 cells (**Fig. 2A**). SUMF-1 acts as a formylglycine modifying enzyme (FGE), converting the critical cysteine residue in ARSB to a formylglycine. Hypoxia produced no decline in SUMF-1 content in the BEC or NCM460 cells. Silencing SUMF-1 by siRNA (**Fig. 2B**) significantly reduced the ARSB activity in the NCM460 cells (**Fig. 2C**) (p<0.001). Maximum reduction in ARSB activity was achieved by the combination of SUMF-1 silencing and hypoxia (p<0.001) (**Fig. 2C**), consistent with requirements for molecular oxygen and formylglycine modification for ARSB activity.

### Hypoxia reduces total sulfated glycosaminoglycans and chondroitin-4-sulfate

**Figure 3:**
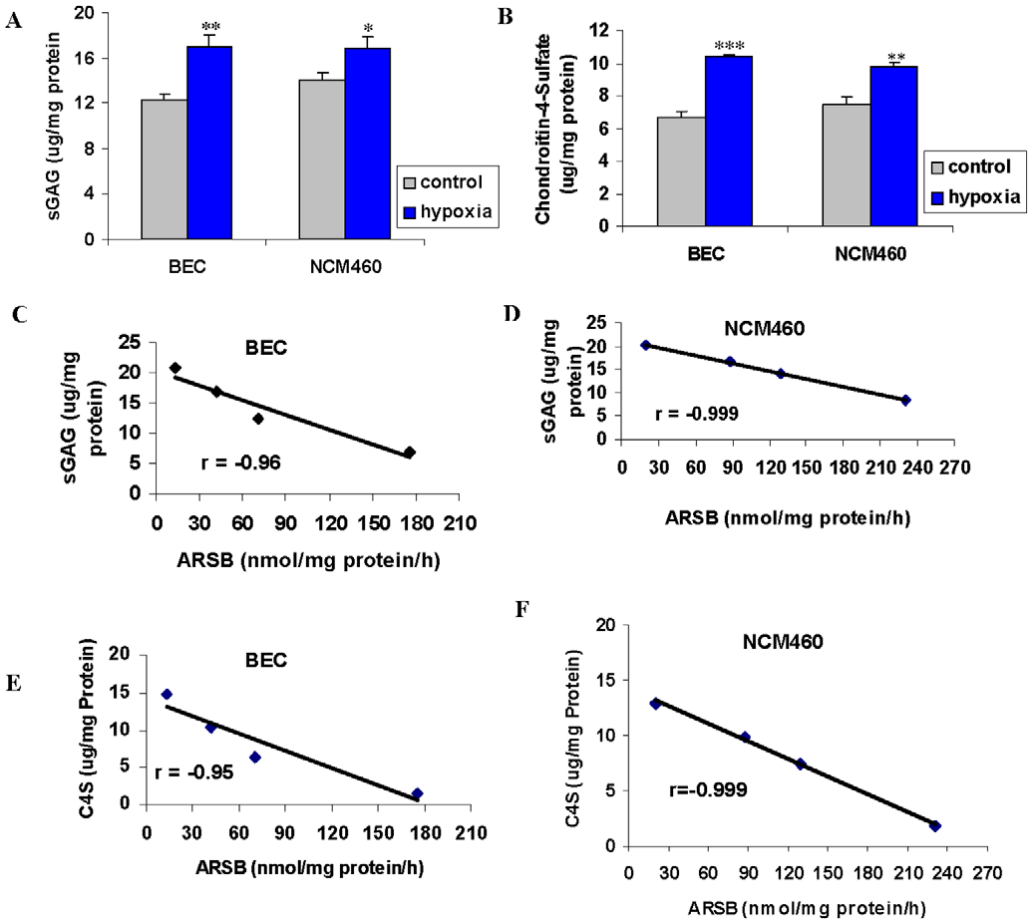
Total sGAG and C4S increased by hypoxia in BEC and NCM460 cells. **A.** In the BEC and the NCM460 cells, the total sGAG increased significantly following 10% O_2_×24 h (p = 0.002, p = 0.013 respectively, unpaired t-test, two tailed), consistent with an effect on ARSB function. **B.** The majority of the increase in sGAG level following 10% O_2_×24 h was attributable to the increases in C4S in the BEC and the NCM460 cells (p<0.001, p = 0.002, respectively). **C, D.** Highly significant inverse correlations were present between the sGAG content and the ARSB activity, using measurements of sGAG and ARSB at baseline, following ARSB knockdown by siRNA, following hypoxia (10% O_2_×24 h), and following ARSB overexpression in the BEC (r = −0.96) and NCM460 cells (r = −0.999). **E, F.** Similar highly significant inverse correlations were present between the C4S content and the ARSB activity in the BEC (r = −0.95) and NCM460 cells (r = −0.99). [sGAG = sulfated glycosaminoglycan; BEC = bronchial epithelial cells; C4S = chondroitin-4-sulfate].

To determine if hypoxia affected cellular sulfated glycosaminoglycans (GAG) or chondroitin-4-sulfate (C4S) content, total cell-associated sulfated GAGs and C4S were measured following exposure of the BEC and the NCM460 cells to 10% O_2_ for 24 hours. Sulfatases, galactosidases, and chondroitinases are involved in sulfated GAG degradation, but no effect of hypoxia on GAG abundance was anticipated. However, in the hypoxic condition, total sulfated GAGs increased from 12.3±0.5 µg/mg protein to 16.9±1.0 µg/mg protein in the BEC (p = 0.002, unpaired t-test, two-tailed) and from 14.0 µg/mg protein to 16.8±0.9 µg/mg protein in the NCM460 cells (p = 0.013, unpaired t-test, two-tailed) (**Fig. 3A**). These increases were mainly attributable to increase in C4S levels, and consistent with decline in ARSB activity leading to inhibition of removal of sulfate groups and inhibition of subsequent degradation. In the BEC, C4S increased from 6.7±0.3 to 10.4±0.1 µg/mg protein (p<0.001, unpaired t-test, two-tailed) and from 7.5±0.4 µg/mg protein to 9.8±0.3 µg/mg protein in the NCM460 cells (p = 0.002, unpaired t-test, two-tailed) (**Fig. 3B**).

The hypoxia-induced increases in total sulfated GAG and C4S content correlate with previously reported changes that followed ARSB silencing and overexpression in the BEC and NCM460 cells and are proportionate to the hypoxia-induced decline in ARSB activity. The cellular sulfated GAG and C4S content are inversely and linearly related to the ARSB activity, including at baseline, and following hypoxia, silencing, and overexpression in the BEC and the NCM460 cells with highly significant inverse correlation (r = −0.99) (**Fig. 3C, 3D**). Similar correlations (r = −0.99) were observed for C4S in the BEC (**Fig. 3E**) and in the NCM460 cells (**Fig. 3F**) in relation to the ARSB activity.

### ARSB silencing increases and ARSB overexpression reduces the activation and expression of HIF-1α

**Figure 4:**
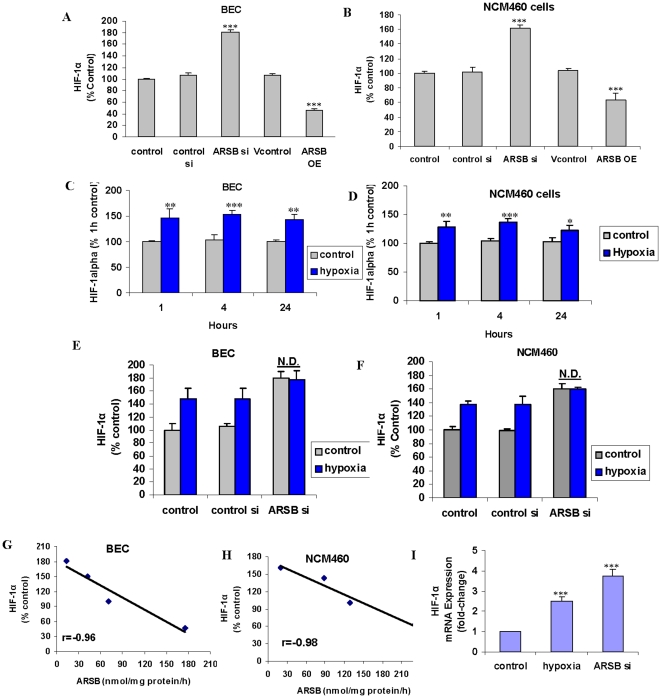
Nuclear HIF-1α levels were increased by ARSB silencing and reduced by ARSB overexpression in BEC and NCM460 cells, and HIF-1α mRNA expression was increased by ARSB silencing in BEC. **A.** Activation of HIF-1α was significantly increased by silencing ARSB and significantly reduced by overexpression of ARSB in the BEC (p<0.001). **B.** In the NMC460 cells, ARSB silencing significantly increased the activation of HIF-1α and overexpression significantly reduced HIF-1α activation in the NCM460 cells (p<0.001). **C.** In the BEC, the effects of hypoxia (10% O_2_) on HIF-1α activation were significant at 1 h (p<0.01), 4 h (p<0.001), and 24 h (p<0.01), with the maximum increase at 4 h. **D.** As in the BEC, in the NCM460 cells, the effects of hypoxia (10% O_2_) on HIF-1α activation were significant at 1 h (p<0.01), 4 h (p<0.001) and 24 h (p<0.05), with the maximum increase at 4 h. **E.** In the BEC, the combined effects of ARSB silencing and hypoxia (10% O_2_) for 24 h had no greater effect on activation of HIF-1α than ARSB silencing alone. **F.** Similarly, in the NCM460 cells, the combined effects of ARSB silencing and hypoxia (10% O_2_) for 24 h had no greater effect on the activation of HIF-1α than ARSB silencing alone. **G, H.** The ARSB activity at baseline, following ARSB overexpression, following ARSB silencing by siRNA, and following hypoxia (10% O_2_)×24 h were inversely correlated with the nuclear HIF-1α levels in the BEC and the NCM460 cells (r = −0.96; r = −0.98, respectively). **I.** HIF-1α mRNA expression increased to 2.5 (±0.23) times the baseline following 10% oxygen×4 h and to 3.73 (±0.34) times the baseline following ARSB silencing by siRNA×24 h (p<0.001) in the BEC. [ARSB = arylsulfatase B; BEC = bronchial epithelial cell; HIF = hypoxia inducible factor].

To assess if some cellular effects of oxygen might be mediated by changes in ARSB activity, measurements of HIF-1α following ARSB silencing and overexpression were performed. When ARSB was silenced by siRNA in the BEC, nuclear HIF-1α increased to ∼181% of baseline. Following ARSB overexpression, nuclear HIF-1α declined to ∼46% of baseline (p<0.001) (**Fig. 4A**). Similarly, nuclear HIF-1α increased following ARSB silencing in the NCM460 cells, and ARSB overexpression produced significant decline in HIF-1α (p<0.001) (**Fig. 4B**). The increases in activated, nuclear HIF-1α in the BEC and the NCM460 cells following ARSB silencing were similar at 1 and 4 hours, but the increases were less at 24 hours (**Fig. 4C, 4D**). The increases in activated HIF-1α were greater following ARSB silencing than from exposure to 10% O_2_ for 1–24 hr.

The combination of ARSB silencing and hypoxia had no greater impact on stimulating HIF-1α than ARSB silencing alone in the BEC (**Fig. 4E**) or in the NCM460 cells (**Fig. 4F**). The relationship between nuclear HIF-1α and ARSB activity, including at baseline and following hypoxia, silencing, and overexpression, reveals a direct, inverse correlation between ARSB activity and nuclear HIF-1α in the BEC (r = −0.96) and in the NCM460 cells (r = −0.98) (**Fig. 4G, 4H**).

mRNA expression of HIF-1α in the BEC was determined by PCR and indicated an increase to 2.5±0.2 times the control following exposure to hypoxia (10% O_2_×4 h) and to 3.7±0.3 times the control siRNA following ARSB silencing ×24 h (p<0.001) (**Fig. 4I**).

### Both hypoxia and ARSB silencing decrease the reduced glutathione/glutathione disulfide ratio and total cellular sulfhydryl content

The unexpected replication by ARSB of the effects of oxygen on HIF-1α suggested that ARSB might act to mediate effects of oxygen through a broad range of cellular effects. We proceeded to assess the impact of hypoxia and ARSB silencing on the overall cellular redox status in the epithelial cells by examining the ratio of reduced glutathione to glutathione disulfide (GSH/GSSG) and the cellular sulfhydryl content.

**Figure 5:**
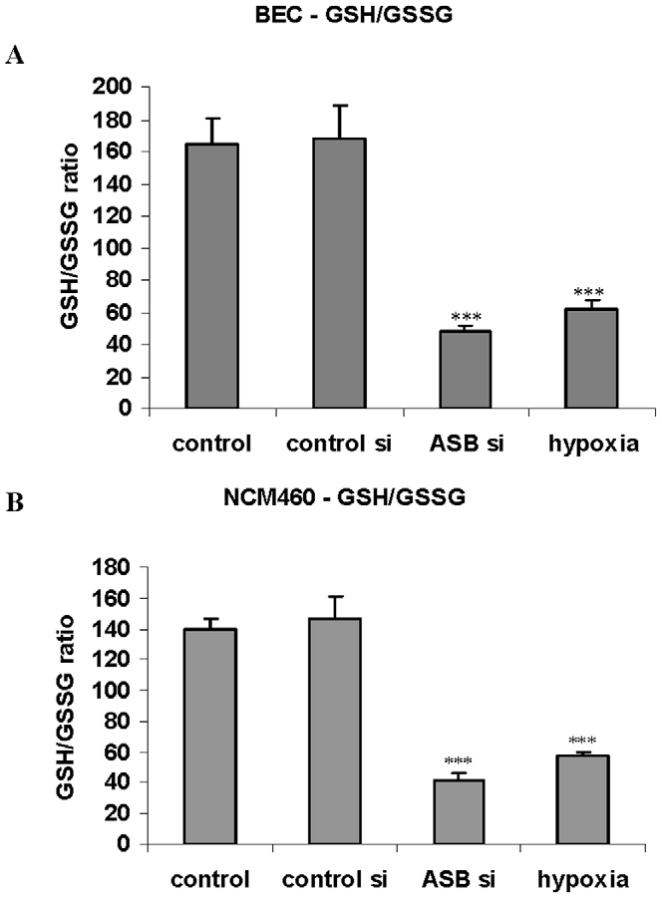
ARSB silencing and hypoxia reduce the GSH/GSSG ratio. **A.** In the BEC, the ratio of reduced glutathione (GSH) to glutathione disulfide declined significantly following hypoxia (10% O_2_×4 h) and following ARSB silencing by siRNA (p<0.001). These effects are consistent with decline in the overall reduced status of the cells. **B.** Similarly, in the NCM460 cells, the reduced glutathione to glutathione disulfide was markedly reduced, attributable to decline in the reduced glutathione (p<0.001) following 10% O_2_×4 h. [BEC = bronchial epithelial cell; ARSB = arylsulfatase B].

In the human bronchial and colonic epithelial cells, both exposure to the reduced oxygen environment (10% O_2_×4 h) and ARSB silencing produced declines in the GSH/GSSG ratio (**Fig. 5A, 5B**). In the BEC, the GSSG content increased to 0.60±0.04 nmol/mg protein from 0.24±0.02 nmol/mg protein, with an associated decline in GSH. Similarly, in the NCM460 cells, the GSSG content increased to 0.46±0.02 nmol/mg protein from 0.21±0.02 nmol/mg protein, and the GSH content declined following exposure to hypoxia.

When ARSB was silenced, greater increases in GSSG in the BEC (to 0.72±0.07 nmol/mg protein) and in the NCM460 cells (to 0.59±0.04 nmol/mg protein) occurred, with greater declines in GSH content and in the GSH/GSSG ratio. These effects demonstrate similarities between the impact of hypoxia and ARSB silencing on the cellular redox state, as manifested by changes in GSH and G-S-S-G.

**Figure 6:**
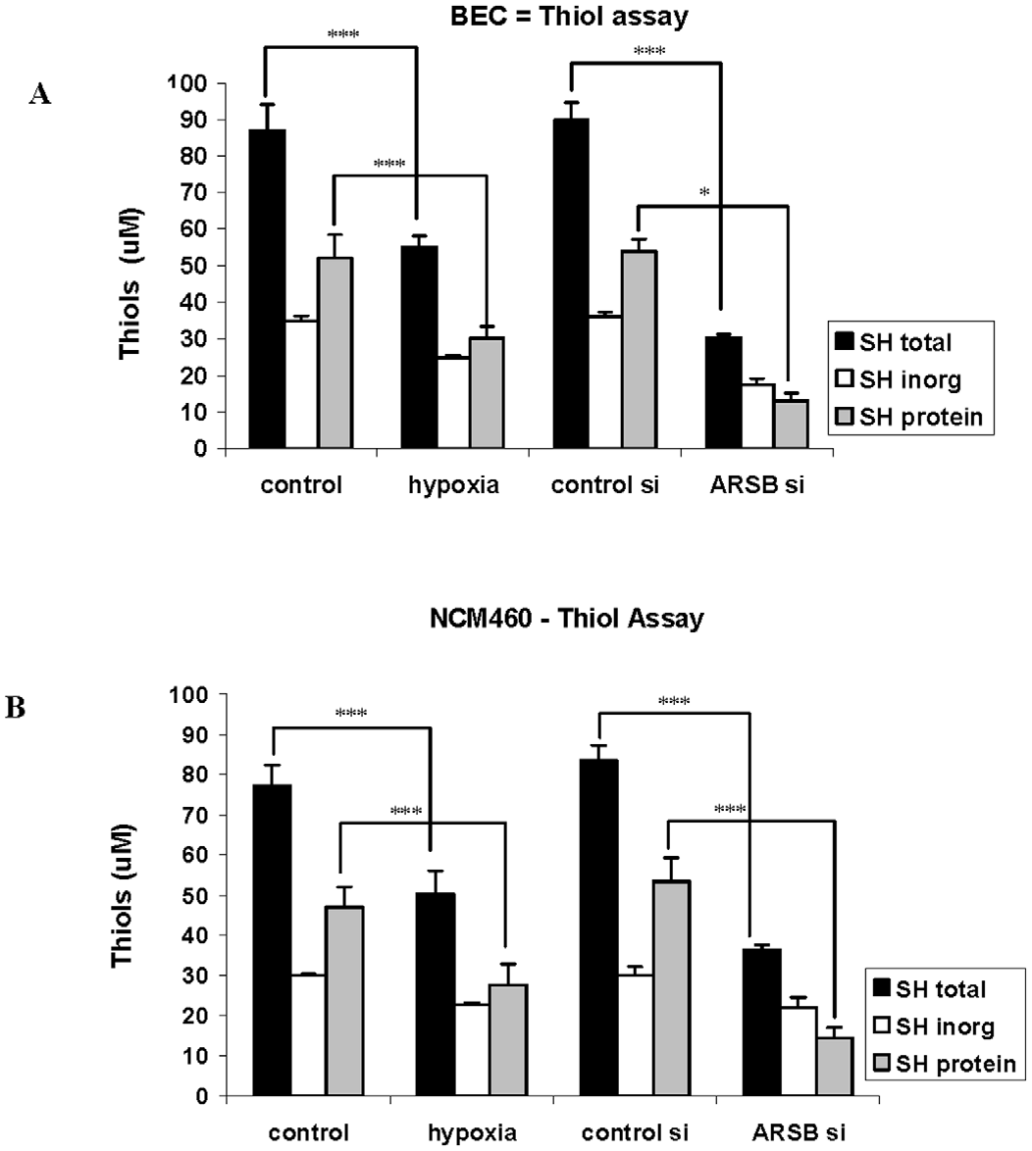
ARSB silencing and hypoxia reduce the total cellular sulfhydryl content. **A.** Overall decline in the total cellular sulfhydryl content in the BEC followed both hypoxia (10% O_2_×4 h) and ARSB silencing, consistent with the observed effect on the reduced glutathione and the overall change in the cellular redox state (p<0.001). Total sulfhydryl content is composed of inorganic and protein thiols, and the inorganic thiol did not change significantly, indicating that the change in total thiols was attributable to the protein-associated thiols. **B.** Similar decline in the thiol cellular sulfhydryl content occurred in the NCM460 cells following either hypoxia (10% O_2_×4 h) or ARSB silencing (p<0.001). The inorganic thiol content did not change significantly, indicating that the change occurred in the protein thiol component. [BEC = bronchial epithelial cell; ARSB = arylsulfatase B].

To further assess how changes in ARSB activity and in oxygenation affected overall cellular redox status, measurements of the total cellular sulfhydryl content, composed of protein-associated and inorganic thiols, were also performed. ARSB knockdown in the BEC and NCM460 cells produced marked declines in the total cell thiol content (p<0.001), attributable to a decline in the protein-associated sulfhydryls, since the declines in the measured inorganic sulfhydryl content were not significant (**Fig. 6A, 6B**). These findings are consistent with the declines in the GSH/GSSG ratio. Exposure of the cells to the hypoxic environment produced a smaller reduction in the total thiol content than ARSB silencing, but proportionate to the hypoxia-induced decline in the ARSB activity.

### Hypoxia and ARSB silencing have similar effects on expression of genes in hypoxia signaling array

**Figure 7:**
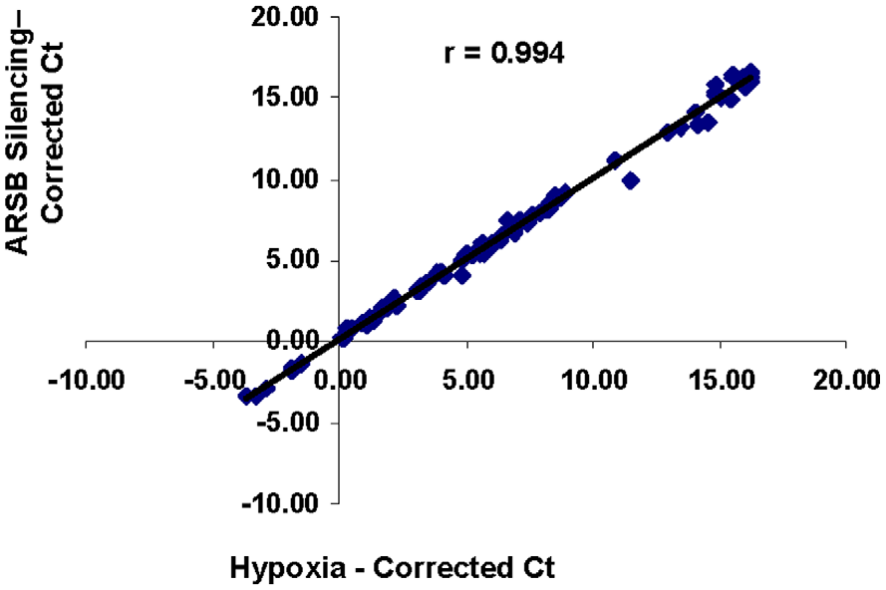
Correlation between Ct values of hypoxia and ARSB silencing for 84 hypoxia-associated genes and 5 housekeeping genes in NCM460 cells. The average corrected Ct values for the hypoxic (10% O_2_×4 h) cells and the ARSB silenced cells had a correlation coefficient r = 0.994. The direction and extent of change in mRNA expression was similar for each of the genes, using simultaneous determinations performed in triplicate on the PCR arrays.

Due to the unexpected and consistent similarities between the effects of hypoxia and ARSB knockdown, the impact of hypoxia and ARSB silencing on gene expression in a hypoxia signaling PCR array was addressed to assess the extent of congruity between the effects of hypoxia and ARSB knockdown. PCR was performed using a hypoxia-signaling array that assayed expression of 84 genes associated with hypoxia and 5 housekeeping genes. Cycle threshold values were averaged using determinations from three replicate arrays and corrected for housekeeping genes. Comparison of the average cycle threshold values between the ARSB silenced and hypoxic results demonstrated that in all cases the direction (increase or decrease) and extent of change in expression were similar between the effects of hypoxia and ARSB silencing, as determined by average cycle threshold values, following correction by the housekeeping genes (r = 0.99; see **Table S1**) (**Fig. 7**). Of the genes evaluated, six had significant p-values (p≤0.05) and fold-changes ≥1.6 (**Table 1**) following both hypoxia and ARSB silencing, when compared to control or scrambled control siRNA, respectively. The affected genes included: interleukin-6 (IL-6); pentraxin-related gene (PTX3); collagen, type 1 alpha 1 (COL1A1); procollagen-lysine, 2-oxoglutarate 5-dioxygenase 3 (PLOD3); plasminogen activator, urokinase (PLAU); and heme oxygenase 1 (HMOX1). Several other genes, including HIF-1α, also had increased expression, but fold-change was less than 1.6.

**Table 1:** Genes with expression significantly increased by both hypoxia and ARSB silencing.

		P-value	Fold change	P-value	Fold change
COL1A1	Collagen, type I, alpha 1	0.031	1.7	0.0046	2.0
HMOX1	Heme oxygenase (decycling) 1	0.030	1.6	0.052	1.7
IL6	Interleukin 6 (interferon, beta-2	0.0023	9.3	0.0047	5.5
PLAU	Plasminogen activator, urokinase	0.016	2.0	0.0040	2.5
PLOD3	Procollagen-lysine, 2-oxoglutarate 5-dioxygenase 3	0.022	1.6	0.0097	2.2
PTX3	Pentraxin-related gene, rapidly induced by IL-1 beta	0.013	3.3	0.0030	5.2

### Galectin-3 binding to C4S declined following hypoxia and ARSB silencing

**Figure 8:**
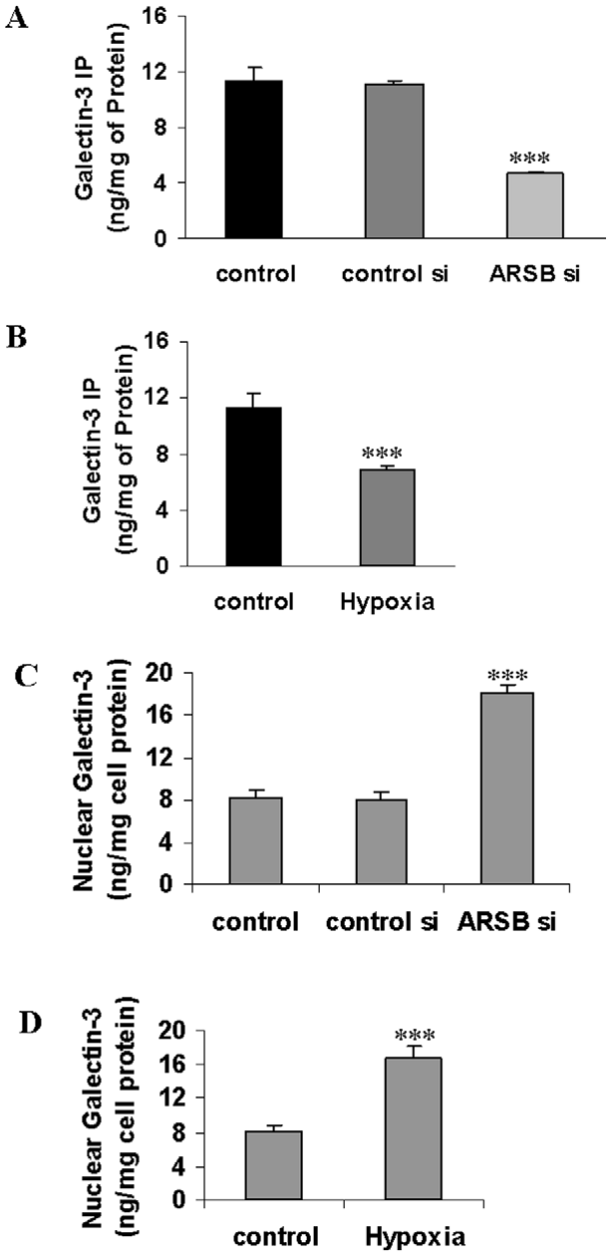
Galectin-3 co-immunoprecipitated with C4S declined following hypoxia and ARSB silencing, and nuclear galectin-3 increased. **A, B.** The galectin-3 that co-immunoprecipitated with C4S in the BEC declined significantly following both hypoxia (10% O_2_×4 h) and ARSB silencing (p<0.001), consistent with reduced association of galectin-3 with more highly sulfated C4S. **C, D.** Galectin-3 content in the nuclear fraction of the BEC increased significantly following both ARSB silencing and hypoxia (10% O_2_×4 h) (p<0.001). [BEC = bronchial epithelial cell; ARSB = arylsulfatase B].

Multiple changes in gene expression followed both hypoxia or ARSB silencing, but a molecular mechanism to explain these effects was undetermined. The only mechanistic action of ARSB is to remove sulfate groups from N-acetylgalactosamine-4-sulfate residues, so transcriptional effects of ARSB silencing must be related to the inhibition of this function. Preferential binding of three members (galectins-3, -7, and -9) of a beta-galactoside-binding protein family to desulfated vs. sulfated GAG has been reported [26]. We hypothesized that galectin-3 binding to C4S might be reduced when ARSB activity was less and chondroitin-4-sulfation was increased. Measurements of galectin-3 co-immunoprecipitated with C4S were performed by ELISA following ARSB silencing and hypoxia (10% O_2_×4 h). Galectin-3 declined from ∼11.3 ng/mg protein to 4.6±0.2 ng/mg protein (p<0.001) following ARSB silencing for 24 h (**Fig. 8A**) and to 6.9±0.3 ng/mg protein (p<0.001) following hypoxia (**Fig. 8B**), consistent with reduced galectin-3 binding to the more highly sulfated C4S. The nuclear galectin-3 increased following both ARSB silencing (**Fig. 8C**) and hypoxia (**Fig. 8D**) in the BEC.

### Proposed mechanism for transcriptional effect of oxygen and ARSB on HIF-1α by galectin-3 and AP-1

**Figure 9:**
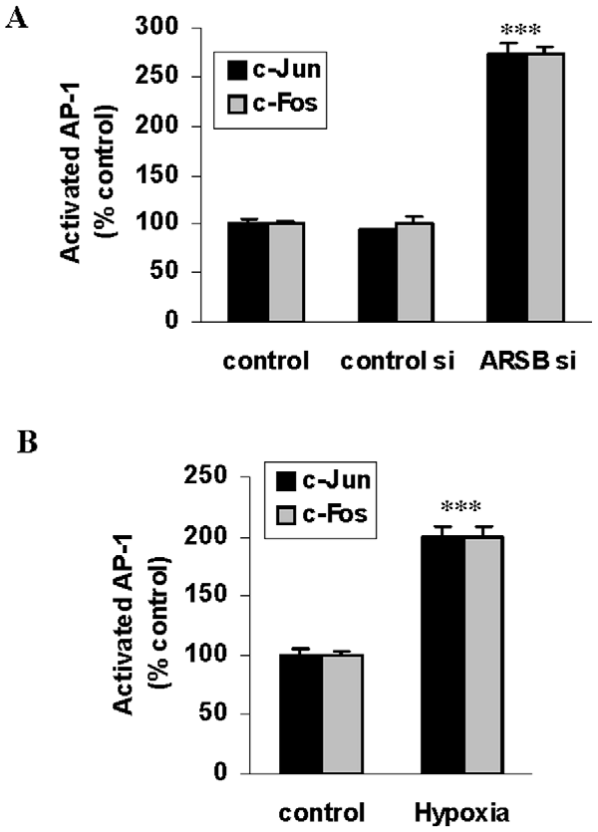
Activated AP-1 increased following ARSB silencing or hypoxia in the BEC. **A**. Nuclear c-Jun significantly increased following ARSB silencing or 10% O_2_×4 h. **B.** Similarly, c-Fos significantly increased following ARSB silencing or 10% O_2_×4 h. [BEC = bronchial epithelial cell; ARSB = arylsulfatase B].

Galectin-3 was considered to be involved in the transcriptional events that followed hypoxia and ARSB silencing, since its binding to C4S and nuclear localization were significantly modified. The effects of galectin-3 have been associated with interactions with AP-1 components c-Jun and c-Fos, and AP-1 binding sites are present on the HIF-1α promoter, suggesting that ARSB silencing and hypoxia might induce transcriptional events through galectin-3 and AP-1. ELISA assays demonstrated significant increases in nuclear c-Jun (**Fig. 9A**) and c-Fos (**Fig. 9B**) (p<0.001), suggesting a molecular mechanism involving AP-1 and galectin-3 by which hypoxia and the associated decline in ARSB activity and increase in chondroitin-4-sulfation might regulate transcriptional activation of HIF-1α.

## Discussion

The study findings indicate that ARSB silencing replicates the effects of hypoxia and are consistent with the requirement for molecular oxygen to activate ARSB. Furthermore, the results suggest that ARSB may mediate intracellular oxygen signaling through effects on chondroitin-4- sulfation. Under normoxic conditions, ARSB silencing increased HIF-1α activation and expression, and ARSB overexpression reduced HIF-1α in human bronchial and colonic epithelial cells, demonstrating the ability of ARSB to regulate HIF-1α activation and expression. In addition to similar effects on HIF-1α activation and expression, hypoxia and ARSB silencing have similar effects, including 1) increase in cellular sulfated GAG and C4S content, 2) decline in GSH/GSSG ratio and total sulfhydryl level, 3) increase or decrease in mRNA expression of 84 hypoxia-associated genes in a PCR array, 4) increase in galectin-3 nuclear localization, and 5) AP-1 activation. These effects suggest a central role for ARSB in the regulation of vital cellular processes.

In addition to the findings that indicate which ARSB silencing can mediate and replicate the effects of hypoxia, the study results provide evidence to explain how the transcriptional effects of hypoxia and ARSB silencing might be mediated. Reduced binding of galectin-3 to C4S following reduction of ARSB activity was demonstrated by decline in the amount of galectin-3 that co-immunoprecipitated with C4S following hypoxia or ARSB knockdown. Decline in binding to more highly sulfated C4S was associated with increased nuclear galectin-3. Increases in nuclear AP-1 components c-Fos and c-Jun were also shown following ARSB silencing and hypoxia. Galectin-3 has been reported to induce transcriptional effects on MUC2 in association with AP-1 in human colon cancer cells [37], and we propose that the effects of hypoxia and ARSB silencing on the activation of HIF-1α may be achieved through the AP-1 binding sites present in the HIF-1α promoter in the presence of the increased nuclear galectin-3 [38]. Additional effects of ARSB, as well as of oxygen, may be mediated through expression of HIF-1α, which has a broad repertoire of known effects, including impact on cell proliferation and epithelial-mesenchymal transition [39], [40]. Galectin-3, like p53 and c-Myc, has a nuclear localization motif and interacts with importins α and β for nuclear translocation and has a wide range of effects on vital cellular processes [41], [42]. The binding of other galectins to C4S may also be modified by changes in chondroitin sulfation when ARSB activity is reduced [28].

**Figure 10:**
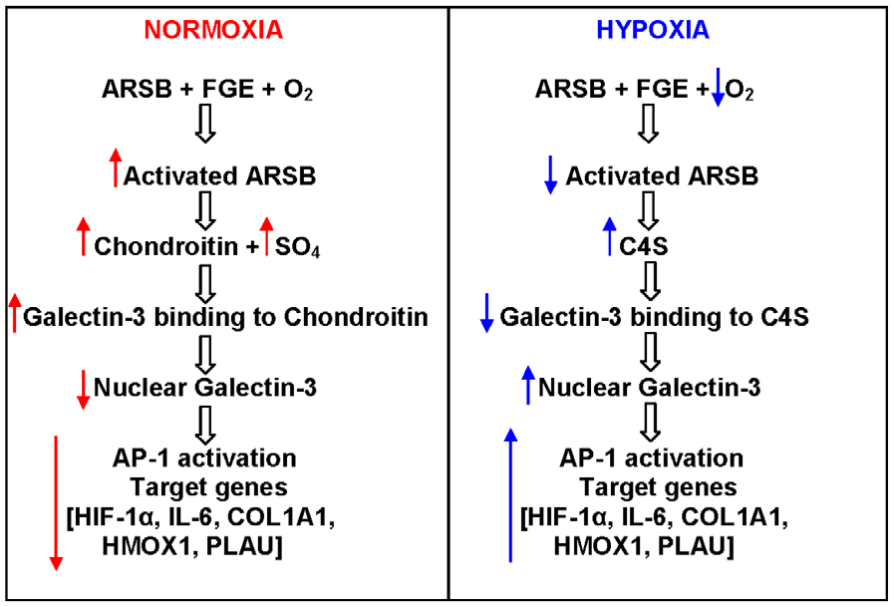
Schematic illustration of oxygen→ARSB→C4S→galectin-3→AP-1→HIF-1α signaling pathway. Normoxic and hypoxic conditions lead to differences in signaling, attributable to reduced ARSB activity leading to increase in chondroitin-4-sulfation, reduced galectin-3 binding to the more highly sulfated C4S, increased nuclear translocalization of galectin-3, and increased transcriptional events in association with activation of AP-1, including increased expression of HIF-1α. [ARSB = arylsulfatase B; FGE = formylglycine (oxoalanine) generating enzyme; C4S = chondroitin-4-sulfate].

**Figure 10** is a schematic representation of the signaling in normoxic and hypoxic pathways, mediated through changes in ARSB activity. Transcriptional events following hypoxia may proceed from reduced ARSB activity, thereby modulating chondroitin-4-sulfation, galectin-3 binding to chondroitin-4-sulfate, galectin-3 nuclear translocation, and AP-1 activation. Variation in the chondroitin-4-sulfation interaction with galectin-3 affects the availability of galectin-3 for nuclear translocation and subsequent transcriptional events involving interaction of galectin-3 with AP-1. The promoters for IL-6, HMOX1, PLAU, COL1A1, genes significantly upregulated by both hypoxia and ARSB silencing in the hypoxia PCR array (**Table 1**), have AP-1 binding sites, indicating a transcriptional mechanism by which they also may be upregulated. The impact of changes in GAG sulfation on nuclear translocation of oncogenes and other transcription factors is a subject for further study which may have significant implications for how genes are regulated by extra-nuclear, and even extracellular, events.

In protists, yeasts, and plants, a sulfate assimilation pathway (SAP) has been described by which sulfate is progressively reduced, leading to production of glutathione, cysteine, and methionine [29]–[36]. The SAP involves adenosine 5′-phosphosulfate (APS) and 3′-phosphoadenosine 5′-phosphosulfate (PAPS). In mammalian cells, PAPS synthetases (PAPSS1 and PAPSS2) have been identified that combine the APS and PAPS functions and activate sulfate [43], [44]. The current study findings demonstrate that diminished availability of sulfate due to reduced ARSB activity following hypoxia in the human epithelial cells leads to decline in the GSH/GSSG ratio and the sulfhydryl content, indicating a profound impact on the overall cellular redox status. This suggests the operation of a mechanism by which molecular oxygen can regulate sulfate assimilation through activation of ARSB in mammalian epithelial cells. The cascade of reactions from sulfate to reduced sulfur in the sulfate assimilation pathway (SAP) (also known as the sulfate activation complex) can be invoked to integrate oxygen signaling, sulfate metabolism, and redox status, since the cascade of reactions from sulfate to reduced sulfur would be impaired due to reduced availability of sulfate following inhibition of ARSB activity and reduced production of free sulfate. The bacterial SAP involves interaction with several critical enzymes, including thioredoxin and glutaredoxin that act as hydrogen donors for PAPS reductase [34], and is thermodynamically driven by GTP hydrolysis [35], [36]

Effects on glutathione reduction impact on other complex redox processes, such as those of monothiol glutaredoxins that utilize reduced glutathione and a cysteine residue to bind to a bridging [2Fe-2S] cluster [45]. Decreased ratio of reduced glutathione to glutathione disulfide is associated with selective promotion of disulfide bond formation within cytoplasmic proteins, leading to functional impairment [46]. For normal cellular aerobic metabolism, coenzyme A (CoA), a sulfhydryl, is processed to the thioester acetyl CoA. The production of acetyl-CoA from pyruvate by the pyruvate dehydrogenase complex is inhibited by increase in the ratio of acetyl-CoA to CoA, reflecting the impact of a thiol on regulation of aerobic metabolism [47]. Impairment of sulfate assimilation, due to reduced activity of ARSB, in association with reduced sulfhydryl content, would impair oxidative metabolism by inhibition of pyruvate dehydrogenase. This suggests a mechanism whereby impairment of aerobic metabolism, as hypothesized by Warburg [48], might follow from decline in ARSB activity, and is consistent with reported findings of reduced ARSB activity in malignant cells [1], [3], [22], [23].

The extensive, unexpected replication between the effects of hypoxia and ARSB silencing in the BEC and NCM460 cells suggests that ARSB mediates some intracellular effects of oxygen. In turn, the effects of hypoxia on C4S and sulfated GAG content indicate that a pathway of sulfate metabolism may be regulated by oxygen through activation of ARSB. Effects of both hypoxia and ARSB silencing on the reduced glutathione-glutathione disulfide ratio and on the cellular sulfhydryl levels demonstrate ramifications of reduced availability of free sulfate on the overall cellular redox state. Since removal of the 4-sulfate residue of N-acetylgalactosamine-4-sulfate is the only known direct effect of ARSB, ARSB may act as a redox switch that regulates the sulfate assimilation pathway. Transcriptional effects may also be mediated by ARSB through chondroitin-4-sulfation and the associated changes in galectin binding to more or less highly sulfated GAGs. By integrating the effect of oxygen with 1) chondroitin sulfation, 2) sulfate assimilation and cellular redox status, and 3) transcriptional regulation through variation in the interaction of galectins with GAGs, ARSB is a critical link in our understanding of how fundamental cell chemistry can regulate vital cell processes.

## Materials and Methods

### Culture of human bronchial and colonic epithelial cells

Normal human bronchial epithelial cells with retinoic acid (NHBE; Lonza Walkersville, Inc., Walkersville MD) were grown as recommended using Bronchial Epithelial Cell Growth Media (BEGM; Lonza). NCM460 cells (INCELL, San Antonio, TX), derived from normal human colonic mucosa, were grown as recommended in M3:10A media (INCELL) [49]. Cells were initially grown in 12-well tissue culture clusters at 37°C, in a humidified, 5% CO_2_, normoxic environment, and then were placed in a modular incubator chamber (Billups-Rothenberg, Inc., Del Mar, CA) which was loaded with a mixture of 85% N_2_, 5% CO_2_, and 10% O_2_ for time periods ranging from 15 minutes to 24 hours. The sealed chamber was placed in a humidified incubator at 37°C. For experiments to determine the recovery from hypoxia, cell preparations were returned to the original normoxic conditions for 4 hours, following 4 hours of hypoxia.

### Sulfatase activity assays

Determinations of ARSB activity were performed using a fluorimetric assay with the substrate 4-methylumbilliferyl sulfate (4-MUS) [22]–[24]. 20 µl of cell homogenate or of spent media and 80 µl of assay buffer (0.05 M Na acetate buffer, pH 5.6) were combined with 100 µl of substrate (5 mM 4-MUS in assay buffer) in wells of a microplate. The microplate was incubated for 30 minutes at 37°C. The reaction was stopped by 150 µl of stop buffer (Glycine-Carbonate buffer, pH 10.7), and fluorescence was measured at 360 nm (excitation) and 465 nm (emission) in a microplate reader) (FLUOstar, BMG LABTECH, Inc., Cary, NC). Activity of galactose-6-sulfatase (GALNS), steroid sulfatase (STS), and arylsulfatase A (ARSA) was also measured in the epithelial cells, as previously described [22]–[24].

### ARSB silencing and overexpression

Cell preparations that were subjected to ARSB silencing or overexpression were grown in 12-well clusters. At 60% confluency, cells were transfected with siRNA for ARSB (Qiagen, Valencia, CA) or by control siRNA for 24 hours, as previously detailed [3], [4], [6]. Overexpression was performed with an ARSB construct (OriGene Technologies, Inc., Rockville, MD) as previously described [3], [4], [6], [22].

### Induction of hypoxia

Measurements of pO2 in the cell cultures were performed using an oxygen needle probe (Lazar Research Laboratories, Los Angeles, CA), following exposure to either the normoxic or hypoxic environment at 1 and 4 hours. Confirmation of hypoxic conditions was achieved by these measurements, which indicated decline of ∼40% in the oxygen saturation of the spent media under hypoxic conditions vs. the normoxic control. Selection of 10% O_2_ environment was based on conditions in previous reports indicating that a 50% reduction in the available oxygen produced significant changes, but without severe toxicity [50], [51].

### SUMF-1 assay and SUMF-1 silencing by siRNA

Sulfatase modifying factor (SUMF)-1 in the control and treated cell extracts was detected by ELISA (USCN Life Sciences, Inc., Wuhan, China) that captured cellular SUMF-1 in microtiter wells coated with anti-SUMF-1 monoclonal antibody. SUMF-1 was detected by biotinylated SUMF-1 polyclonal antibody and avidin-horseradish peroxidase (HRP). The enzyme activity of bound HRP was determined by adding hydrogen peroxide-tetramethylbenzidine (TMB) chromogenic substrate. The magnitude of the optical density of the developed color was measured in an ELISA plate reader at 450 nm. SUMF-1 concentrations were determined from a standard curve made with known concentrations of SUMF-1. Sample values were normalized with the total protein concentrations determined by BCA protein assay kit (Pierce, Thermo Fisher Scientific, Rockford, IL). SUMF-1 ELISA was used to detect the extent of silencing following treatment of cells by commercial SUMF-1 siRNA (Qiagen). Four different siRNAs were tested for their effect on SUMF1 expression, and the siRNA prepared against the target sequence 5′-GTCGAGGAGGCCTGCATAATA-3′ was selected for knockdown of SUMF-1 in the cells, following an established protocol for gene silencing [3], [4].

### Measurements of sulfated GAG and C4S levels

Measurements of total sulfated GAG and C4S were performed as previously described [22]. Briefly, the Blyscan™ assay kit (Biocolor Ltd, Newtownabbey, N. Ireland) was used for detection of the sulfated GAG, based on the reaction of 1,9-dimethylmethylene blue with the sulfated oligosaccharides in the GAG chains. C4S content was determined following immunoprecipitation with C4S antibody, as reported previously [22].

### Activated HIF-1α ELISA

Nuclear extracts of treated and control cells were prepared using a nuclear extract kit [Active Motif, Carlsbad, CA]. Activated HIF-1α in the nuclear extract of the control and treated cells was determined by a commercial oligonucleotide-based ELISA (R & D Systems, MN). Activated HIF-1α in the nuclear extract bound to a biotinylated double-stranded oligonucleotide containing a consensus sequence of the HIF-1α binding site, and the HIF-1α-oligonucleotide complexes were captured by an immobilized antibody specific for HIF-1α that was coated onto the wells of a microtiter plate. Captured HIF-1α molecules were subsequently detected by streptavidin-horseradish peroxidase (HRP), and HRP activity was determined by adding hydrogen peroxide-tetramethylbenzidine (TMB) chromogenic substrate. The optical density for the developed color was measured in an ELISA reader (FLUOstar, BMG) at 450 nm, after stopping the reaction with 2N sulfuric acid. The intensity of the developed color was proportionate to the quantity of activated HIF-1α in each sample. The sample values were normalized with the total cell protein and expressed as a percentage of control.

### PCR array for hypoxia-associated gene expression

Hypoxic PCR gene array (SABiosciences, Frederick, MD) to detect changes in expression of 84 hypoxia-associated genes was performed in triplicate using 384-well microplates, in which 4-samples were tested simultaneously for each of the mRNAs of interest. Samples included: NCM460 cells in which ARSB was silenced by siRNA, NCM460 cells treated by control siRNA, untreated NCM460 cells, and NCM460 cells exposed to 10% oxygen for 4 hours. Plates were analyzed by calculations of cycle threshold (Ct) values for each of the wells, and by normalization using the Ct values to controls from five housekeeping genes. Comparisons were made between the mean Ct values (n of 3) for each of the four conditions using the normalized Ct values. Statistical significance was determined by t-test that compared the normalized Ct values for each of the genes of interest. Correlation coefficient (r) was calculated using Excel software.

QPCR was performed for HIF-1α with forward primer 5′-GCTATTTGCGTGTGAGGAAAC-3′ and reverse primer 5′-CACCATCATCTGTGAGAACCA-3′. QPCR was performed using established methods [22].

### Glutathione (GSH/GSSG/Total) assay

GSH, GSSG, and total glutathione in the control and treated cells were determined (Glutathione Detection Kit, BioVision, Inc., Mountain View, CA). Samples were collected on 6N perchloric acid on ice to avoid oxidation of labile GSH. GSH was determined by reaction of cell samples with O-phthalaldehyde (OPA). OPA reacted with cellular GSH, but not GSSG, to generate fluorescence. GSH+GSSH total was determined by first adding a reducing agent which, converted GSSG to GSH, and then reacting with OPA to produce fluorescence. To measure GSSG specifically, a GSH quencher was added initially to remove GSH, preventing the reaction with OPA, but not interacting with GSSG. Reducing agent was then added to destroy excess quencher and to convert GSSG to GSH. The fluorescence was measured in a fluorescence plate reader (FLUOstar) with excitation and emission wavelengths of 340 nm and 420 nm, respectively.

### Total, inorganic, and protein sulfhydryl determinations

Cellular sulfhydryl in the control and treated samples were determined by a commercial assay (Molecular Probes, Eugene, OR) [52], [53]. Inorganic thiols in the samples reduced a disulfide bond, releasing the active enzyme papain from its inactive S-S form. The activity of the enzyme was then measured using the chromogenic papain substrate, *N*-benzoyl-L-arginine, *p*-nitroanilide (L-BAPNA). The intensity of the developed color was proportionate to the quantity of thiols in each sample. To measure the total (inorganic and protein-associated) thiols, cystamine was added to the reaction, which permitted the detection of poorly accessible thiols on proteins with high pKa values. Cystamine, a disulfide, underwent an exchange reaction with protein thiols, yielding 2-mercaptoethylamine (cysteamine), which then released active papain. Active papain then acted on L-BAPNA to develop color, and absorbance was measured in a spectrophotometer at 410 nm. The protein sulfhydryl component was calculated as the difference between the total and the inorganic components.

### Galectin-3 measurements by ELISA in cell fractions and following immunoprecipitation with chondroitin-4-sulfate

Galectin-3 was determined by a sandwich ELISA kit (R&D Systems, Minneapolis, MN) for human galectin-3. The wells of a microtiter plate were coated with specific anti-galectin-3 monoclonal antibody, and nonspecific sites were blocked by a blocking buffer with 1% bovine serum albumin (BSA). Cell fractionation was performed, separating the cytoplasmic and nuclear fractions by centrifugation for 30 seconds at 14,000 g. The galectin-3 from the spent media, cytoplasmic and nuclear fractions were separately captured into the microtiter wells by specific galectin-3 antibody. Captured Galectin-3 was then detected by biotin-conjugated secondary galectin-3 antibody and streptavidin-horseradish peroxidase (HRP). Hydrogen peroxide-tetramethylbenzidine (TMB) chromogenic substrate was used to develop the color, and the intensity of color was measured at 450 nm in an ELISA plate reader (FLUOstar, BMG). The galectin-3 concentrations were extrapolated from a standard curve, and sample values were normalized with total protein content (BCA Protein Assay Kit, Pierce (Rockford, IL). In addition, galectin-3 was measured following immunoprecipitation of the BEC cells with C4S antibody (4D1; Abnova, Novus Biologicals, Littleton, CO). Cell lysates were prepared from treated and control cells. C4S (4D1) antibody binds to native C4S and cross-reactivity with other chondroitin sulfates is not significant [22]. The antibody was added to the cell lysates (1 µg/mg of protein), and tubes were rotated overnight in a shaker at 4°C. Next, 100 µl of pre-washed Protein L-agarose (Santa Cruz Biotechnology) was added to each tube, and the tubes were incubated overnight at 4°C. The Protein L-agarose-treated beads were washed three times with phosphate-buffered saline containing Protease Inhibitor Mixture. The precipitate was eluted with dye-free elution buffer and subjected to galectin-3 assay as described above.

### Detection of nuclear AP-1 by oligonucleotide-based ELISA

Oligonucleotide binding assay (Active Motif, Carlsbad, CA) was used to detect nuclear c-Jun and c-Fos in the BEC following hypoxia (10% oxygen×4 hr) or ARSB silencing by siRNA. Nuclear extracts from treated and control cells were prepared using a nuclear extract preparation kit (Active Motif). Activated AP-1 components in the samples were detected by oligonucleotide-based ELISA. The nuclear extracts were added to the wells of the 96-well microtiter plate, pre-coated with an AP-1 consensus oligonucleotide sequence (5′-TGAGTCA-3′). The AP-1 proteins from the nuclear extract attached to the coated oligonucleotides, and the bound c-Jun and c-Fos were detected by specific antibodies and anti-rabbit-HRP-conjugated IgG. The specificity of the binding of c-Jun and c-Fos with the coated nucleotide sequence was determined by comparison with the binding when a known quantity of free consensus nucleotide or mutated nucleotide was added in the reaction buffer. Colorimetric readout was performed with hydrogen peroxide-TMB chromogenic substrate. After the reaction was stopped with 2N sulfuric acid, the optical density of the developed color was measured in an ELISA plate reader (FLUOstar, BMG) at 450 nm. The intensity of the developed color proportionately represents the quantity of c-Jun or c-Fos in each sample. The sample values were normalized with the total cell protein and expressed as percent of untreated control.

### Statistics

Experiments were performed with at least three independent biological samples and with technical replicates of all measurements. P-values ≤0.05 were considered statistically significant. One-way ANOVA with Tukey-Kramer post-test was performed for analysis of the significance of differences between measurements, unless stated otherwise. For the PCR array, statistical significance was corrected for multiple comparisons, and Ct values were adjusted by normalization with five housekeeping genes.

## Supporting Information

Table S1 **Corrected average cycle threshold (Ct) values of hypoxia-associated genes in PCR array following hypoxia or ARSB silencing.**
(DOC)
