# Supplementary material for: Hypoxia Reduces Arylsulfatase B Activity and Silencing Arylsulfatase B Replicates and Mediates the Effects of Hypoxia
Source: PLoS One. 2012 Mar 13;7(3):e33250. doi: 10.1371/journal.pone.0033250 (PMC3302843; doi:10.1371/journal.pone.0033250)
Supplement: Table S1 — Corrected average cycle threshold (Ct) values of hypoxia-associated genes in PCR array following hypoxia or ARSB silencing. (DOC) [file pone.0033250.s001.doc]

| **Table S1. Corrected average cycle threshold (Ct) values of hypoxia-associated genes in PCR array following hypoxia or ARSB silencing.** |  |  | **AVG ΔC_t_ (Ct(GOI) - Ave Ct (HKG))*** | |
| --- | --- | --- | --- | --- |
| **GeneBank** | **Symbol** | **Description** | **ARSB**  **Silencing** | **Hypoxia** |
| NM_001124 | ADM | Adrenomedullin | 2.05 | 2.22 |
| NM_006412 | AGPAT2 | 1-acylglycerol-3-phosphate O-acyltransferase 2 (lysophosphatidic acidacyltransferase, beta) | 9.62 | 9.46 |
| NM_015239 | AGTPBP1 | ATP/GTP binding protein 1 | 6.3 | 6.33 |
| NM_001039667 | ANGPTL4 | Angiopoietin-like 4 | 8.24 | 8.28 |
| NM_003491 | ARD1A | ARD1 homolog A, N-acetyltransferase (S. cerevisiae) | 3.17 | 3.03 |
| NM_014862 | ARNT2 | Aryl-hydrocarbon receptor nuclear translocator 2 | 15.9 | 14.85 |
| NM_004324 | BAX | BCL2-associated X protein | 4.2 | 3.83 |
| NM_003670 | BHLHE40 | Basic helix-loop-helix family, member e40 | 3.15 | 3.18 |
| NM_001168 | BIRC5 | Baculoviral IAP repeat-containing 5 | 9.24 | 8.35 |
| NM_001738 | CA1 | Carbonic anhydrase I | 12.92 | 12.98 |
| NM_033292 | CASP1 | Caspase 1, apoptosis-related cysteine peptidase (interleukin 1, beta, convertase) | 4.16 | 3.99 |
| NM_001752 | CAT | Catalase | 4.06 | 4.17 |
| NM_001791 | CDC42 | Cell division cycle 42 (GTP binding protein, 25kDa) | 1.37 | 1.24 |
| NM_001275 | CHGA | Chromogranin A (parathyroid secretory protein 1) | 15.15 | 14.85 |
| NM_000088 | COL1A1 | Collagen, type I, alpha 1 | 6.63 | 6.97 |
| NM_004380 | CREBBP | CREB binding protein | 7.11 | 7.05 |
| NM_000100 | CSTB | Cystatin B (stefin B) | -1.62 | -1.84 |
| NM_134268 | CYGB | Cytoglobin | 10.89 | 11.09 |
| NM_001348 | DAPK3 | Death-associated protein kinase 3 | 6.01 | 6 |
| NM_006400 | DCTN2 | Dynactin 2 (p50) | 5.27 | 4.99 |
| NM_001938 | DR1 | Down-regulator of transcription 1, TBP-binding (negative cofactor 2) | 7.33 | 7.39 |
| NM_001397 | ECE1 | Endothelin converting enzyme 1 | 6.95 | 6.97 |
| NM_001402 | EEF1A1 | Eukaryotic translation elongation factor 1 alpha 1 | -3.35 | -3.23 |
| NM_001428 | ENO1 | Enolase 1, (alpha) | 1.12 | 0.84 |
| NM_001429 | EP300 | E1A binding protein p300 | 5.82 | 5.78 |
| NM_001430 | EPAS1 | Endothelial PAS domain protein 1 | 1.35 | 1.26 |
| NM_000799 | EPO | Erythropoietin | 15.73 | 16 |
| NM_017892 | PRPF40A | PRP40 pre-mRNA processing factor 40 homolog A (S. cerevisiae) | 8.84 | 8.68 |
| NM_002067 | GNA11 | Guanine nucleotide binding protein (G protein), alpha 11 (Gq class) | 5.4 | 5.07 |
| NM_000175 | GPI | Glucose phosphate isomerase | 2.07 | 1.83 |
| NM_000581 | GPX1 | Glutathione peroxidase 1 | 0.16 | 0.05 |
| NM_000518 | HBB | Hemoglobin, beta | 15.05 | 15.08 |
| NM_001530 | HIF1A | Hypoxia inducible factor 1, alpha subunit (basic helix-loop-helix transcription factor) | 1.34 | 1.4 |
| NM_017902 | HIF1AN | Hypoxia inducible factor 1, alpha subunit inhibitor | 6.26 | 6.2 |
| NM_152794 | HIF3A | Hypoxia inducible factor 3, alpha subunit | 14.89 | 15.45 |
| NM_000189 | HK2 | Hexokinase 2 | 9.32 | 8.77 |
| NM_002133 | HMOX1 | Heme oxygenase (decycling) 1 | 7.89 | 7.89 |
| NM_006388 | KAT5 | K(lysine) acetyltransferase 5 | 7.5 | 7.46 |
| NM_006389 | HYOU1 | Hypoxia up-regulated 1 | 6.53 | 6.47 |
| NM_000612 | IGF2 | Insulin-like growth factor 2 (somatomedin A) | 13.62 | 13.57 |
| NM_000596 | IGFBP1 | Insulin-like growth factor binding protein 1 | 16.3 | 15.95 |
| NM_000575 | IL1A | Interleukin 1, alpha | 2.95 | 3.7 |
| NM_000600 | IL6 | Interleukin 6 (interferon, beta 2) | 9.99 | 11.47 |
| NM_002184 | IL6ST | Interleukin 6 signal transducer (gp130, oncostatin M receptor) | 5.46 | 5.72 |
| NM_003870 | IQGAP1 | IQ motif containing GTPase activating protein 1 | 2.28 | 2.25 |
| NM_003685 | KHSRP | KH-type splicing regulatory protein | 6.05 | 5.66 |
| NM_000222 | KIT | V-kit Hardy-Zuckerman 4 feline sarcoma viral oncogene homolog | 16.35 | 16.21 |
| NM_002299 | LCT | Lactase | 16.26 | 15.59 |
| NM_000230 | LEP | Leptin | 11.2 | 10.85 |
| NM_000528 | MAN2B1 | Mannosidase, alpha, class 2B, member 1 | 5.52 | 5.33 |
| NM_014484 | MOCS3 | Molybdenum cofactor synthesis 3 | 7.72 | 7.65 |
| NM_005954 | MT3 | Metallothionein 3 | 13.47 | 14.59 |
| NM_002466 | MYBL2 | V-myb myeloblastosis viral oncogene homolog (avian)-like 2 | 6.3 | 5.75 |
| NM_000625 | NOS2 | Nitric oxide synthase 2, inducible | 15.39 | 14.85 |
| NM_017617 | NOTCH1 | Notch homolog 1, translocation-associated (Drosophila) | 7.52 | 6.67 |
| NM_000905 | NPY | Neuropeptide Y | 16.37 | 15.55 |
| NM_001161 | NUDT2 | Nudix (nucleoside diphosphate linked moiety X)-type motif 2 | 5.07 | 4.87 |
| NM_006849 | PDIA2 | Protein disulfide isomerase family A, member 2 | 14.08 | 14.11 |
| NM_003768 | PEA15 | Phosphoprotein enriched in astrocytes 15 | 3.46 | 3.29 |
| NM_015553 | IPCEF1 | Interaction protein for cytohesin exchange factors 1 | 13.41 | 14.21 |
| NM_002658 | PLAU | Plasminogen activator, urokinase | 3.38 | 4.13 |
| NM_001084 | PLOD3 | Procollagen-lysine, 2-oxoglutarate 5-dioxygenase 3 | 6.4 | 6.3 |
| NM_005036 | PPARA | Peroxisome proliferator-activated receptor alpha | 8.13 | 8.24 |
| NM_004156 | PPP2CB | Protein phosphatase 2 (formerly 2A), catalytic subunit, beta isoform | 1.7 | 1.62 |
| NM_006251 | PRKAA1 | Protein kinase, AMP-activated, alpha 1 catalytic subunit | 5.44 | 5.51 |
| NM_002795 | PSMB3 | Proteasome (prosome, macropain) subunit, beta type, 3 | 2 | 1.67 |
| NM_002852 | PTX3 | Pentraxin-related gene, rapidly induced by IL-1 beta | 13.17 | 13.49 |
| NM_000964 | RARA | Retinoic acid receptor, alpha | 9.23 | 8.87 |
| NM_000991 | RPL28 | Ribosomal protein L28 | 7.4 | 7.13 |
| NM_000994 | RPL32 | Ribosomal protein L32 | 1.6 | 1.48 |
| NM_002952 | RPS2 | Ribosomal protein S2 | -3.37 | -3.68 |
| NM_001011 | RPS7 | Ribosomal protein S7 | -1.35 | -1.49 |
| NM_005500 | SAE1 | SUMO1 activating enzyme subunit 1 | 8.99 | 8.5 |
| NM_006516 | SLC2A1 | Solute carrier family 2 (facilitated glucose transporter), member 1 | 2.05 | 1.74 |
| NM_001042 | SLC2A4 | Solute carrier family 2 (facilitated glucose transporter), member 4 | 15.95 | 16.21 |
| NM_003089 | SNRNP70 | Small nuclear ribonucleoprotein 70kDa (U1) | 4.22 | 4.05 |
| NM_003128 | SPTBN1 | Spectrin, beta, non-erythrocytic 1 | 8.81 | 8.61 |
| NM_006396 | SSSCA1 | Sjogren syndrome/scleroderma autoantigen 1 | 8.49 | 8.31 |
| NM_006937 | SUMO2 | SMT3 suppressor of mif two 3 homolog 2 (S. cerevisiae) | 0.56 | 0.28 |
| NM_000360 | TH | Tyrosine hydroxylase | 12.66 | 12.67 |
| NM_003312 | TST | Thiosulfate sulfurtransferase (rhodanese) | 5.33 | 5.28 |
| NM_006000 | TUBA4A | Tubulin, alpha 4a | 1.19 | 0.65 |
| NM_003355 | UCP2 | Uncoupling protein 2 (mitochondrial, proton carrier) | 5.79 | 5.94 |
| NM_003376 | VEGFA | Vascular endothelial growth factor A | 4.1 | 4.89 |
| NM_004048 | B2M | Beta-2-microglobulin | 0.92 | 1.1 |
| NM_000194 | HPRT1 | Hypoxanthine phosphoribosyltransferase 1 | 3.53 | 3.51 |
| NM_012423 | RPL13A | Ribosomal protein L13a | 0.26 | 0.19 |
| NM_002046 | GAPDH | Glyceraldehyde-3-phosphate dehydrogenase | -2.86 | -2.88 |
| NM_001101 | ACTB | Actin, beta | -1.86 | -1.92 |

*GOI = gene of interest; HKG = housekeeping; AVG=average; Ct=cycle threshold;
